# Tamoxifen induces cellular stress in the nervous system by inhibiting cholesterol synthesis

**DOI:** 10.1186/s40478-015-0255-6

**Published:** 2015-11-26

**Authors:** Franziska Denk, Leanne M. Ramer, Erin L. K. S. Erskine, Mohammed A. Nassar, Yury Bogdanov, Massimo Signore, John N. Wood, Stephen B. McMahon, Matt S. Ramer

**Affiliations:** Wolfson Centre for Age-Related Diseases, King’s College London, Guy’s Campus, London, SE1 1UL UK; Djavad Mowafaghian Centre for Brain Health, University of British Columbia, 2215 Wesbrook Mall, Vancouver, V6T 1Z3 BC Canada; Department of Biomedical Science, The University of Sheffield, Western Bank, Sheffield, S10 2TN UK; Wolfson Institute for Biomedical Research (WIBR), University College London (UCL), Cruciform Building, London, WC1E 6BT UK; Institute of Child Health, London, WC1N 1EH UK; International Collaboration On Repair Discoveries, University of British Columbia, Vancouver, V5Z 1 M9 BC Canada

**Keywords:** Dentate gyrus, Olfactory bulb, Activating transcription factor 3, Cholesterol biosynthesis, Sensory neurons

## Abstract

**Background:**

Tamoxifen (TAM) is an important cancer therapeutic and an experimental tool for effecting genetic recombination using the inducible Cre-Lox technique. Despite its widespread use in the clinic and laboratory, we know little about its effects on the nervous system. This is of significant concern because TAM, via unknown mechanisms, induces cognitive impairment in humans. A hallmark of cellular stress is induction of Activating Transcription Factor 3 (*Atf3*), and so to determine whether TAM induces cellular stress in the adult nervous system, we generated a knock-in mouse in which *Atf3* promoter activity drives transcription of TAM-dependent Cre recombinase (Cre-ERT2); when crossed with tdtomato reporter mice, *Atf3* induction results in robust and permanent genetic labeling of cells in which it is up-regulated even transiently.

**Results:**

We found that granular neurons of the olfactory bulb and dentate gyrus, vascular cells and ependymal cells throughout the brain, and peripheral sensory neurons expressed tdtomato in response to TAM treatment. We also show that TAM induced *Atf3* up-regulation through inhibition of cholesterol epoxide hydrolase (ChEH): reporter expression was mitigated by delivery in vitamin E-rich wheat germ oil (vitamin E depletes ChEH substrates), and was partially mimicked by a ChEH-specific inhibitor.

**Conclusions:**

This work demonstrates that TAM stresses cells of the adult central and peripheral nervous systems and highlights concerns about clinical and experimental use of TAM. We propose TAM administration in vitamin E-rich vehicles such as wheat germ oil as a simple remedy.

## Introduction

Tamoxifen (TAM), a “selective estrogen receptor modulator” (SERM), is among the most widely-used anti-cancer drugs for women with estrogen receptor-positive breast tumors. Although its side effects have long been considered minor, there is accumulating evidence that a subpopulation of TAM-treated patients experience cognitive disturbances such as confusion, deterioration of verbal memory, and executive function such as decision-making, symptoms often referred to as “TAM brain fog” [1–4]. Changes in human brain structure and metabolism with TAM treatment have also been reported [5]. Yet, there has been surprisingly little work done in animal models to identify TAM-induced changes in structure or function of the nervous system (either central or peripheral) [6], although in one study it was associated with a loss of glial progenitor cells in the corpus callosum and with reduced cell division in neurogenic regions of the CNS [7].

The dearth of preclinical work on potential adverse effects of TAM is concerning not only because of its reported cognitive effects in humans, but also because TAM is a widely-used tool for manipulating gene expression in vivo via the inducible Cre-Lox technique. Cre recombinase is a bacteriophage enzyme, which can remove sequences of DNA that lie between engineered loxP sites (referred to as “floxed” sequences). By attaching Cre to a mutated estrogen receptor (ERT2, which binds TAM but not endogenous estrogens; Cre-ERT2), it is possible to control precisely when a DNA sequence can be excised. As an example, mice expressing Cre-ERT2 can be crossed with another transgenic line containing a floxed “stop” signal preceding the sequence for a fluorescent reporter. The resulting mice express Cre-ERT2, which is restricted to the cytoplasm. When it binds TAM, Cre-ERT2 is translocated to the nucleus, where it can excise the stop signal, resulting in reporter expression. This technique has been used in mice to permanently label newly-generated neurons in the hippocampus [8] and transiently-active neurons following exposure to novel environments [9].

What remains little-acknowledged in studies using the Cre-ERT2 system is that TAM acts at the endogenous estrogen receptor α (ERα), hence its clinical use in cancer. Furthermore, TAM binds other endogenous molecules, including sigma 1 and 2 receptors on the endoplasmic reticulum [10], and cholesterol epoxide hydrolase (ChEH) – an enzyme complex involved in cholesterol biosynthesis [11]. In fact, the affinity of TAM for ChEH is second only to that for ERα, which may underlie its counter-intuitive efficacy in ER-negative breast cancers [11]. TAM thus has the potential to alter neurophysiology via any one of these avenues.

We generated a knock-in mouse expressing CreERT2 driven by the native promoter for activating transcription factor 3 (Atf3), one of a number of transcription factors upregulated by cellular stress, such as the unfolded protein response, tyrosine kinase receptor activation, glutamate receptor hyperactivation, and cellular injury [12]. Crossing the ATF3-CreERT2 line with floxed stop ROSA-tdtomato line, when combined with TAM treatment, allows for permanent labelling of cells even transiently stressed by TAM. Here we report TAM-induced recombination in mature granular neurons of the olfactory bulb and dentate gyrus, populations of neurons involved in memory formation and recall. Recombination also occurred in larger-diameter proprioceptive neurons of the dorsal root ganglion, and in vascular endothelial and smooth muscle cells, and ependyma throughout the brain and spinal cord. We also provide evidence that TAM’s induction of Atf3 expression occurs via ChEH binding, and not via ERα or sigma 1/2 receptors.

## Material and methods

### Generation of mice

**Fig. 1:**
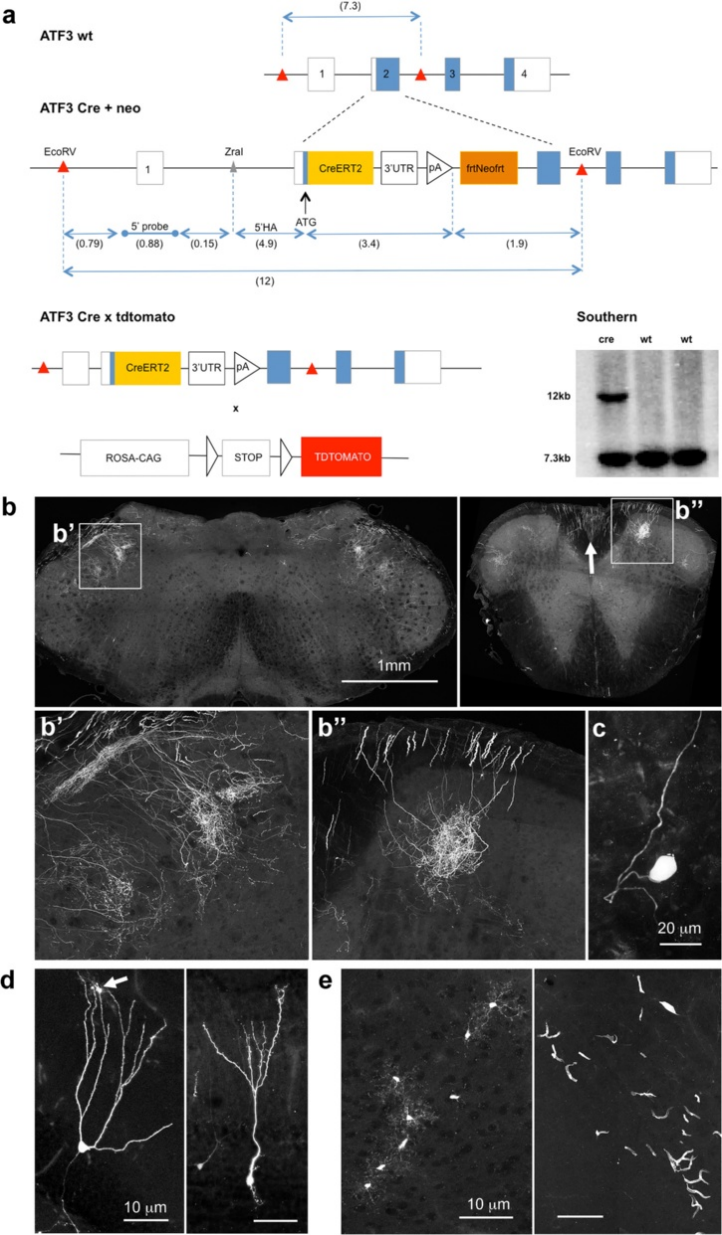
Characterization the naïve ATF3-CreERT2:stop^fl/fl^tdtomato mouse. **a** Genetic strategy used to generate the ATF3 CreERT2 mouse. The wild-type ATF3 locus (ATF3 wt) was modified to generate a transgenic construct (ATF3 Cre + neo), in which CreERT2 was inserted immediately after the ATG start codon. Numbers in brackets designate length of fragments in kilobases. The construct was electroporated into 129SvEv mouse embryonic stem cells and positive clones were selected via neomycin (neo) and identified using Southern blots: three clones are shown as an example. Positive founders were bred with a flp-expressing line to remove the neomycin cassette, and the final ATF3 Cre mice were crossed with a ROSA-tdtomato line to yield an ATF3CreERT2 reporter mouse (ATF3 Cre x tdtomato). Schematics are not drawn to scale. **b** Terminals of trigeminal (left) and DRG axons (right) in the brainstem and upper cervical cord, respectively (regions in boxes enlarged in **b’** and **b”**). The arrow indicates sensory axons in the dorsal funiculus. All images are 30 μm-thick confocal orthogonal projections. **c** A tdtomato-positive sensory neuron from a cervical DRG (42 μm-thick z-projection). **d** Rare recombined granular neurons from the dentate gyrus (left) and olfactory bulb (right) (30 μm-thick z-projections). **e** Occasional clusters (probably clonal) of microglia (left, here from the cerebellum) and vascular endothelial cells (right, in this case from the midbrain) (30 μm-thick z-projections)

The mouse *Atf3* gene was subcloned from a 129 bacterial artificial chromosome (BAC) (bMQ-293K18) into pBluescript (approx. 5 kb of sequence either side of the transcriptional start site in exon 2). Homologous recombination in bacteria was used to insert a construct directly after the ATG start codon containing: Cre fused to the mutated estrogen-ligand binding domain (CreERT2), 1.2 kb of *Atf3* 3′ untranslated region, a Simian virus 40 stop signal and a neomycin cassette flanked by two FLP-recognition target sites. The final targeting vector was sequenced, linearised using a ZraI digest and electroporated into 129 mouse embryonic stem (ES) cells. Positive clones were identified using Southern blotting after digest with EcoRV (Fig. 1a) and injected into blastocysts. The ES cell manipulations and blastocyst injections were carried out by the Transgenic Services of the Institute of Child Health at University College London. After breeding out the neomycin resistance gene from founders using Flp recombinase mice, the main mouse line was generated and is maintained in a heterozygous state (ATF3-CreERT2). The ATF3-CreERT2 mice were crossed with a floxed stop ROSA-tdtomato line (AI14, Jackson Labs) [13] for characterization of expression. They are maintained on a mixed background of 129SvEv and C57BL/6J.

We also used a BAC transgenic mouse in which the promoter for advillin, expressed in all dorsal root ganglion (DRG) neurons, drives CreERT2 [14], and crossed it with the same reporter line as above. For all experiments, mice in treatment and control groups were sex and age-matched.

### Drug treatments

All of the drugs used, their doses, and final animal numbers in each experiment are listed in Table 1. TAM was delivered at a dose of 75 mg/kg intraperitoneal (i.p.) in multiple vehicles, containing varying amounts of α-tocopherol (vitamin E), which prevents accumulation of cholesterol epoxides. Sunflower oil (SFO), which is relatively low in vitamin E (40 mg/100 g), was used as a TAM vehicle and compared with wheat germ oil (WGO), which is relatively rich in vitamin E (~150 mg/100 g) [15]. In some experiments, we added vitamin E to SFO; vitamin E was dissolved in SFO at a concentration of 4.47 mg/ml, to match the dose contained in WGO, chosen based on previous efficacy and toxicity studies of vitamin E in mice [16]. When delivered in wheat germ oil (WGO) or sunflower oil (SFO) with vitamin E, we used a volume of 0.25 ml or 0.5 ml, and gave additional injections of oil or oil and vitamin E alone on the day before and the day after TAM treatment.

**Table 1:** Compounds and doses used in N number of mice

Compound	Dose/ Route of administration	*N*
Tamoxifen in sunflower oil	75 mg/kg, i.p. (1 × day or 3 × days)	5
Tamoxifen in wheat germ oil	75 mg/kg, i.p. (1 × day)	4
Vitamin E	4.47 mg/ml in sunflower oil, i.p.	6
ICI 182,780 (ICI)	20 mg/kg, gavage	3
4,4′,4″-(4-Propyl-[1H]-pyrazole-1,3,5-triyl) trisphenol (PPT)	10 mg/kg, s.c.	3
1,3-di-o-tolylguanidine (DTG)	5 mg/kg, i.p.	3
Diethyl-2-[4-(phenylmethyl) phenoxy]ethanamine hydrochloride (DPPE)	50 mg/kg, i.p.	3

In experiments designed to identify the mechanism of TAM-induced ATF-3 up-regulation, we treated mice with either the anti-estrogen ICI 182,780 (ICI), a “pure” anti-estrogen [17], 4,4′,4″-(4-Propyl-[1*H*]-pyrazole-1,3,5-triyl)*tris*phenol (PPT), a potent ERα agonist [18], or ditolylguanidine (DTG), a sigma-1 and −2 receptor agonist. ICI 182,780 in SFO (20 μg) [19] or SFO only was administered to nerve-injured mice by gavage the day before, the day of, and the day following TAM (or oil only) treatment. 4,4′,4″-(4-Propyl-[1*H*]-pyrazole-1,3,5-triyl)*tris*phenol (PPT), was dissolved in 50 % DMSO in PBS and given by s.c. injection at 10 mg/kg [18] following the same schedule as with ICI 182,780. The sigma receptor agonist 1,3-di-o-tolylguanidine (DTG) was prepared as previously described [20] and delivered by i.p. injection in the same manner as the above drugs. The selective ChEG ligand N,N-Diethyl-2-[4-(phenylmethyl) phenoxy]ethanamine hydrochloride (DPPE, also known as tesmilifene) was administered at a one-time dose of 50 mg/kg i.p. in saline [21]. Control mice received saline-only injections. For treatments with ICI 182,780, PPT, DTG, DPPE, and their controls, mice were killed on the fourth day following treatment.

### Tissue processing and analysis

Mice were deeply anesthetized with sodium pentobarbital (Euthanal) and perfused transcardially with phosphate buffered saline followed by 4 % paraformaldehyde in 0.1 M phosphate buffer (PB). Tissues were dissected and transferred to 20 % sucrose in 0.1 M PB. Brains and spinal cords were embedded in gelatin and cut coronally on a vibratome (100 μm), or were frozen and cut at 50 μm on a cryostat (for co-localization studies). Whole dorsal root ganglia or 16 μm-thick cryosections were processed for immunohistochemistry. For DRG whole-mounts and brain sections, quantification was carried out on ~1 μm-thick confocal sections imaged with a Zeiss LSM 710 confocal system. DRG cryosections were imaged with a Zeiss AxioObserver Z.1 equipped with a Yokogawa spinning disk. In the dentate gyrus and olfactory bulb we measured the density of recombined neurons in the granular layers of each structure (3 sections per animal at the level of the median eminence), or counted recombined neurons (for DPPE studies). In the DRG we traced recombined profiles and used recursive translation [22] to convert profile distributions (in which large profiles are numerically overrepresented and lead to overestimations of the number of small diameter profiles) to cell distributions.

To quantify size-frequency distributions of all DRG neurons, we used cervical DRG whole-mounts from advillin-CreERT2 mice treated for 3 days with 75 mg/kg TAM in SFO, and killed on the fourth day. Cell densities between SFO and WGO groups were compared using Student’s *t*-test. Proportions of recombined neurons in the DRG were compared with a one-way analysis of variance (ANOVA) followed by a post-hoc Holm-Sidak test for pairwise differences. The Kolmogorov-Smirnov goodness-of-fit test was used to determine whether cell size-frequency distributions differed significantly.

### Antibodies

The following antibodies were used: rabbit anti-ATF3 (1:200, Santa Cruz Biotechnology Inc.), mouse anti-neurofilament 200 (NF200; clone N52, 1:500, Sigma), rabbit anti-calcitonin gene related peptide (CGRP; 1:4000, Sigma), rabbit anti-tyrosine hydroxylase (TH; 1:1000, Millipore). To identify non-peptidergic nociceptors, slides were first incubated in isolectin B4 (IB4;1:400, Sigma) followed by an anti-IB4 primary antibody (1:2000, Vector laboratories). To characterize recombined neurons in the brain we used mouse anti-Neuronal Nuclei (NeuN; 1:100, Millipore) and rabbit anti-calbindin d28k (1:1000, Swant). Secondary antibodies included donkey anti-rabbit Alexa-488, donkey anti-goat Alexa-647, donkey anti-mouse Dylight 650 (all at 1:1000, Invitrogen), donkey anti-mouse aminomethylcoumarin (AMCA; 1:100, Jackson labs). Nuclear counterstains were 4′,6-diamidino-2-phenylindole (DAPI) (in ProLong Gold coverslipping medium, Invitrogen) or Hoechst 33342 (1:10,000, Sigma).

## Results

### Generation and characterization of ATF3-CreERT2:stop^fl/fl^tdtomato mice

A transgenic knock-in strategy was used to replace the endogenous Atf3 locus with an ATF3-CreERT2 allele (Fig. 1a). The resulting mice, even in a homozygous state, which effectively constitutes an *Atf3* knock-out, appear phenotypically normal and outwardly indistinguishable from their wild-type littermates. The ATF3-CreERT2 line was then crossed with a ROSA-flox-stop-tdtomato line to obtain a permanent reporter of *Atf3* activity.

When examining the resulting mice for tdtomato signal, it became clear that the ATF3-CreERT2 construct displays a small degree of TAM-independent “leakiness”. Under normal circumstances, Atf3 is expressed in very few regions of the adult nervous system [12]. We and others have found that a small number of uninjured sensory neurons express Atf3 [23], and evidence of rare spontaneous recombination was found in reporter-expressing trigeminal and spinal sensory neurons, and their terminals in the spinal trigeminal nucleus and spinal cord (Fig. 1b, c). Allen Brain Atlas in situ hybridization data [24] show ATF3 expression only in granular neurons of the olfactory bulb and dentate gyrus, and accordingly, in naïve ATF3-CreERT2:stop^fl/fl^tdtomato mice, occasional reporter-expressing neurons were found in both of these regions (Fig. 1d). We also found occasional clusters (probably clones) of microglia and vascular endothelial cells throughout the brain (Fig. 1e). As would be expected in the case of spontaneous Atf3 activation, recombination events increased with age, ranging from 3 to 5 cells per DRG in 8 week old mice to 10–20 cells in 7 month old mice (data not shown).

### TAM-induced Atf3 induction in cortical regions

**Fig. 2:**
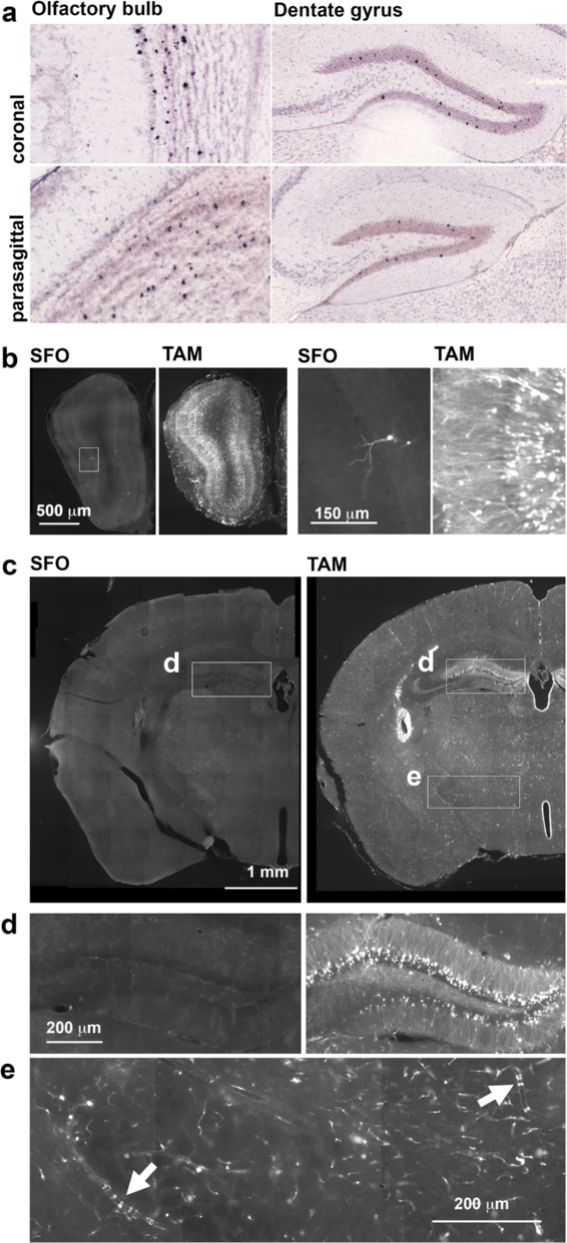
**a** Allen Brain Atlas in situ hybridization studies of ATF3 mRNA expression in dentate gyrus and olfactory bulb. Sections are 25 μm thick. **b** In the absence of TAM, a few neurons have undergone recombination in the granular layer of the OB; with TAM, recombination is widespread and is far more than expected from Allen Brain Atlas ATF3 in situ hybridization studies. Insets are shown enlarged on the right. **c–e** TAM induces recombination in DG granule neurons, ependymal cells of the cerebral ventricles, in endothelial cells and in vascular smooth muscle cells (arrows in **e**). All sections (except those from the Allen Institute) are 30 μm thick and are single-plane images taken on a standard epifluorescence microscope. TAM: TAM. DG: dentate gyrus. OB: olfactory bulb. Image credit **a**: Allen Institute for Brain Science

**Fig. 3:**
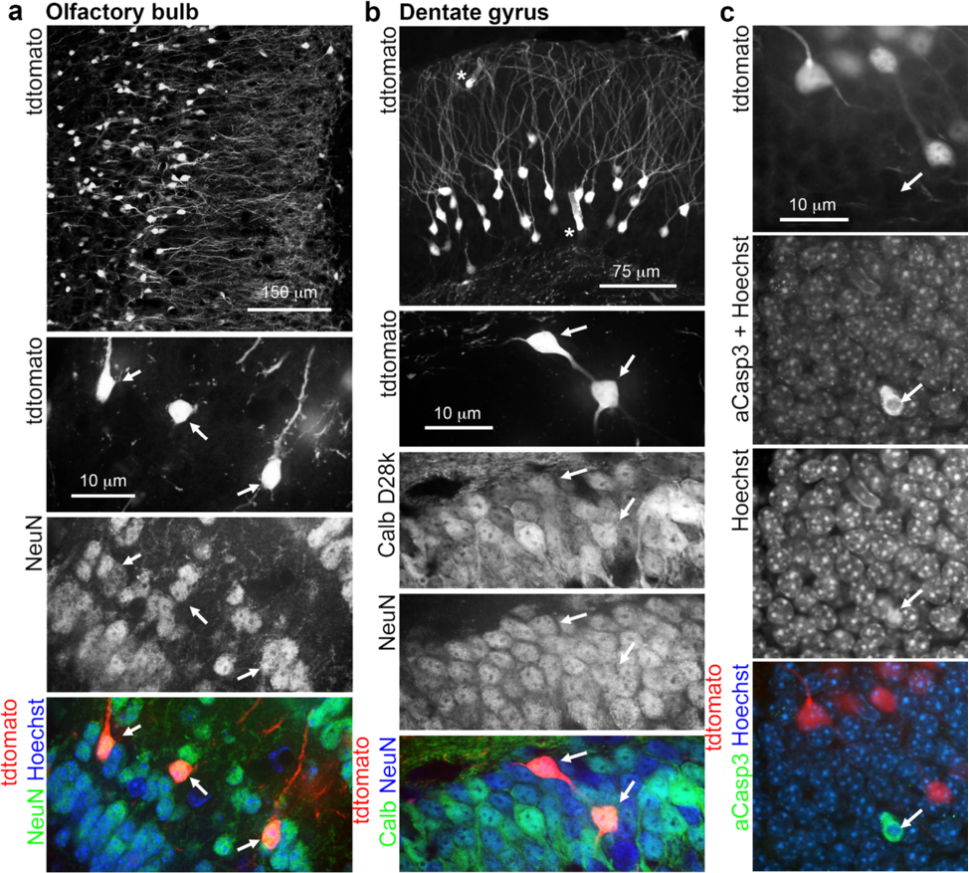
Characterization of recombined cells in the dentate olfactory bulb and dentate gyrus. **a** Olfactory bulb. recombined cells are neuronal, as is evident from morphology and colocalization with NeuN (arrows). **b** Dentate gyrus. Here too, tdtomato-expressing cells express NeuN and the dentate granule cell lineage marker calbindin D28k (Calb D28k). Asterisks indicate recombined endothelial cells. **c** Recombined neurons do not undergo cell death. Very few cells in either the olfactory bulb or dentate gyrus expressed the pro-apoptotic marker activated caspase 3 (aCasp3) or had condensed chromatin, and these were never tdtomato-positive. Shown is an example of a rare dying neuron from the dentate gyrus

Since TAM is known to have side effects of a cognitive nature in cancer patients, we sought to investigate its effects in relevant regions of the brain. The Allen Brain Atlas [24] suggests that *Atf3* is very sparsely expressed in the brain, with only some labelling in the granule cell layers of the olfactory bulb and dentate gurys (Fig. 2a). Indeed in vehicle-treated ATF3-CreERT2:stop^fl/fl^tdtomato animals, an occasional Atf3 positive cell was present in these areas due to the leakiness of the ERT2 construct (Figs. 2b and 1d). TAM administration resulted in wide-spread recombination in granular neurons of the olfactory bulb and dentate gyrus (Fig. 2b-d). Recombination also occurred throughout the brain and spinal cord in vascular endothelial, smooth muscle and ependymal cells (Fig. 2e), but not in tanycytes lining the ventral part of the 3^rd^ ventricle (data not shown). In the olfactory bulb and dentate gyrus, neuronal expression of reporter was confirmed by colocolization of tdtomato with NeuN (olfactory bulb) and NeuN/calbindin D28k (dentate gyrus) (Fig. 3a, b). Reporter expression was not simply due to TAM-induced nuclear translocation of the ATF3-CreERT2 construct in cells expressing Atf3 as the density of tdtomato-expressing neurons was far higher than would be expected based on *Atf3* in situ hybridization data. Atf3 upregulation was not associated with increased expression of pro-apoptotic activated caspase 3: there were occasional dying neurons in both regions, but these were never also tdtomato-positive (Fig. 3c).

### TAM-induced Atf3 induction in primary afferent neurons

**Fig. 4:**
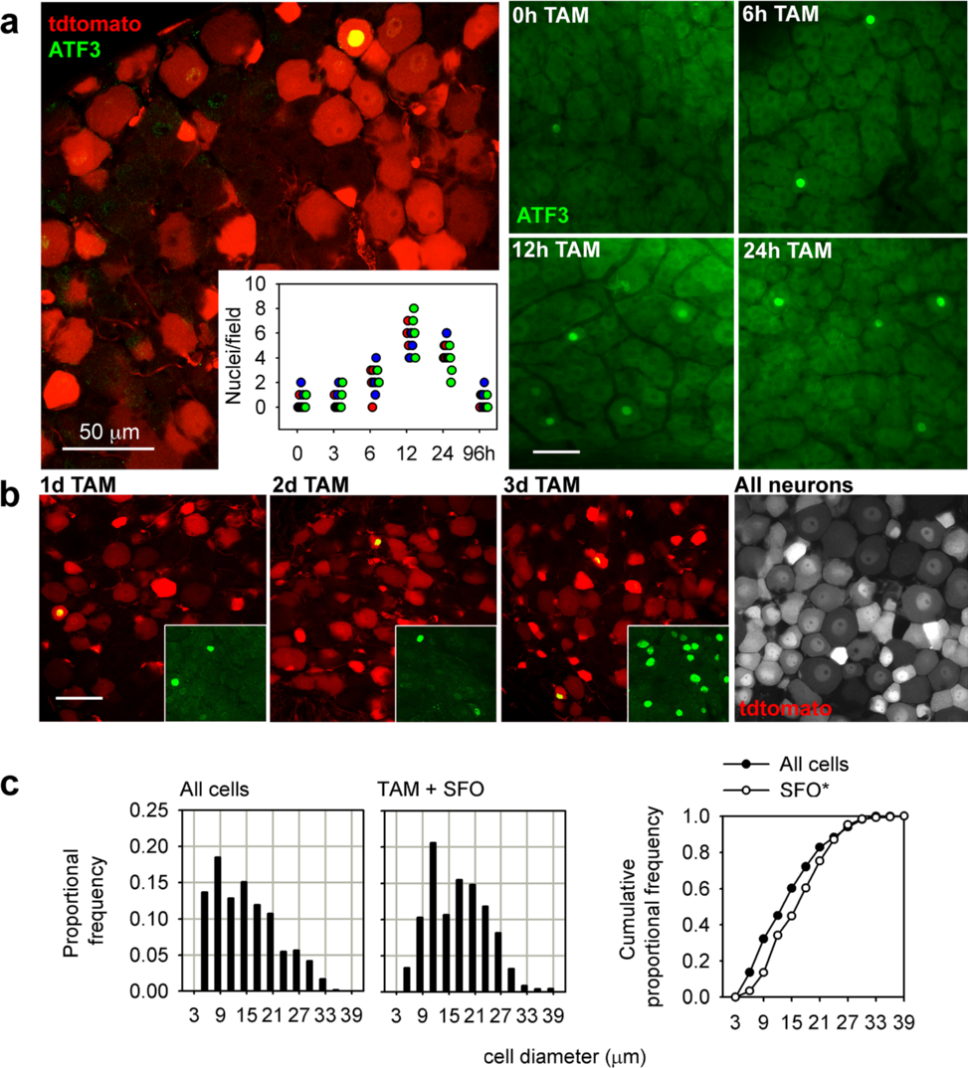
TAM (TAM) induces transient ATF3 upregulation and recombination in the DRG. **a** (left), tdtomato and ATF3 expression from a cervical DRG, 4 days after a single 75 mg/kg TAM injection (single 1 μm-thick confocal plane). **a** (right), ATF3 expression in the trigeminal ganglion at various timepoints following TAM (single optical planes). The inset in shows quantitative differences in the number of ATF3-positive nuclei per 400 × 400 μm single optical section. Each point represents a single image (four each from three animals per timepoint). **b** The proportion of neurons undergoing recombination after TAM does not increase with repeated daily dosing. Main panels are 1 μm-thick optical sections, insets are 30 μm-thick stacks from the same fields of view. The far-right image is from a TAM-treated advillin-CreERT2:stop^fl/fl^tdtomato mouse, in which recombination occurs in nearly all DRG neurons. **c** Size-frequency distribution analysis reveals that after TAM treatment, recombined neurons tend to be larger (there is a rightward shift in the distributions *p* < 0.05, Kolmogorov-Smirnov goodness-of-fit test). SFO: sunflower oil, the TAM vehicle

TAM also induced high levels of tdtomato expression in the DRG (Fig. 4). More than 20 % of neurons were affected, even after a single intraperitoneal dose of TAM at 75 mg/kg. Using an antibody to stain for Atf3, we could show that noticeably elevated nuclear expression was transient (Fig. 4a). Repeated dosing of mice with TAM did not increase the number of recombined neurons in the DRG beyond that which occurred after a single 75 mg/kg i.p. injection (Fig. 4b), although the number of ATF3-positive neuronal nuclei was noticeably increased 24 h after a third daily injection, indicating that TAM repeatedly stresses otherwise healthy neurons.

**Fig. 5:**
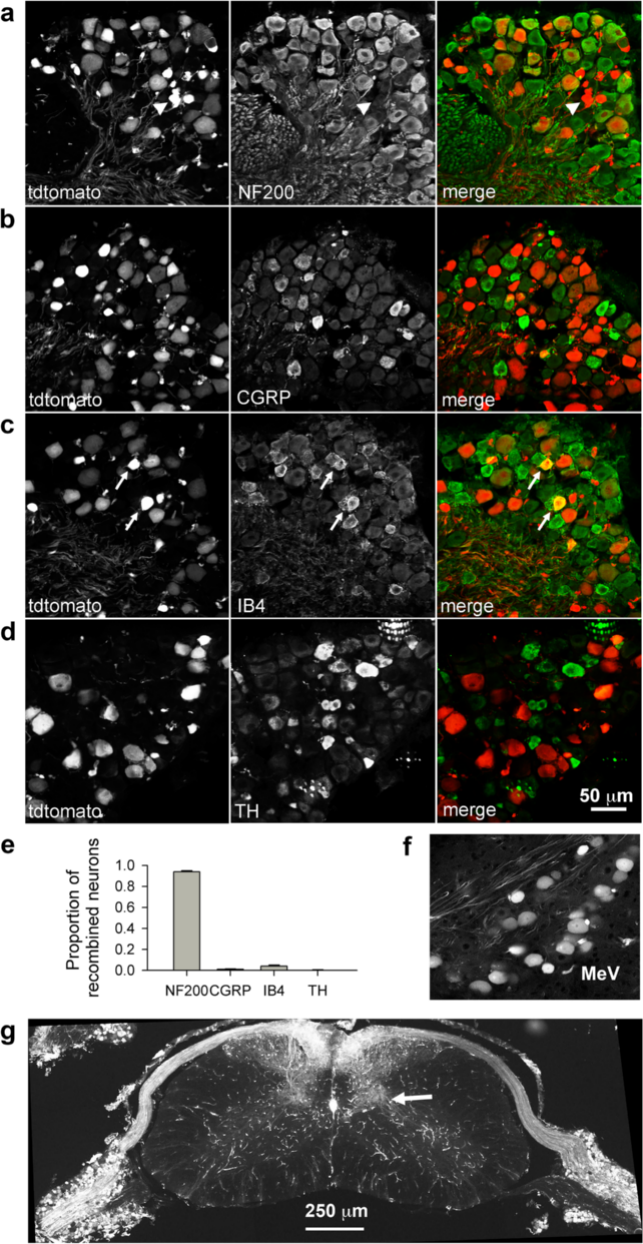
Phenotypic distribution of ATF3-expressing DRG neurons after TAM treatment. **a** NF200, expressed by large diameter mechanosensory DRG neurons. Arrowhead indicates a few NF200-negative profiles. **b** CGRP, expressed by peptidergic nociceptors. **c** IB4 lectin binding by non-peptidergic nociceptors, doubly-labeled cells are indicated by arrows. **d** TH expression by small diameter DRG neurons. **e** Quantification of marker expression by tdtomato-positive neurons. The vast majority of recombined DRG neurons express NF200. **f** Trigeminal mesencephalic proprioceptive neurons also undergo recombination with TAM treatment. **g** Central projections of recombined neurons are most dense in the ascending dorsal columns and in Clarke’s column (arrow). Other recombined structures are vascular endothelial and smooth muscle cells and ependymal cells (see also Fig. 2). All images are single confocal planes

TAM-induced tdtomato expression occurred preferentially in large diameter neurons, indicating that they may be more vulnerable to cellular stress. This was confirmed using analysis of cell size diameter (Fig. 4c), as well as co-staining with markers for the four prominent mouse DRG subpopulations [25] (Fig. 5): neurofilament 200 (NF200) for large diameter mechanosensitive neurons; calcitonin gene-related peptide (CGRP) and isolectin B4 (IB4) for peptidergic and non-peptidergic nociceptors, respectively; and tyrosine hydroxylase (TH) for a fourth small diameter population (Fig. 5a–d). The vast majority of recombination occurred in NF200 positive neurons, with a small percentage also in non-peptidergic IB4 positive nociceptors (Fig. 5e). Other cell populations found to be preferentially affected by TAM treatment were mesencephalic trigeminal neurons that convey proprioceptive information from the masseter (Fig. 5f), as well as vascular endothelial, smooth muscle and ependymal cells (Fig. 5g). In the spinal cord, primary afferent projections to Clarke’s column (a proprioceptive relay to the cerebellum) were particularly dense.

### ChEH inhibition accounts for Atf3 induction by TAM

**Fig. 6:**
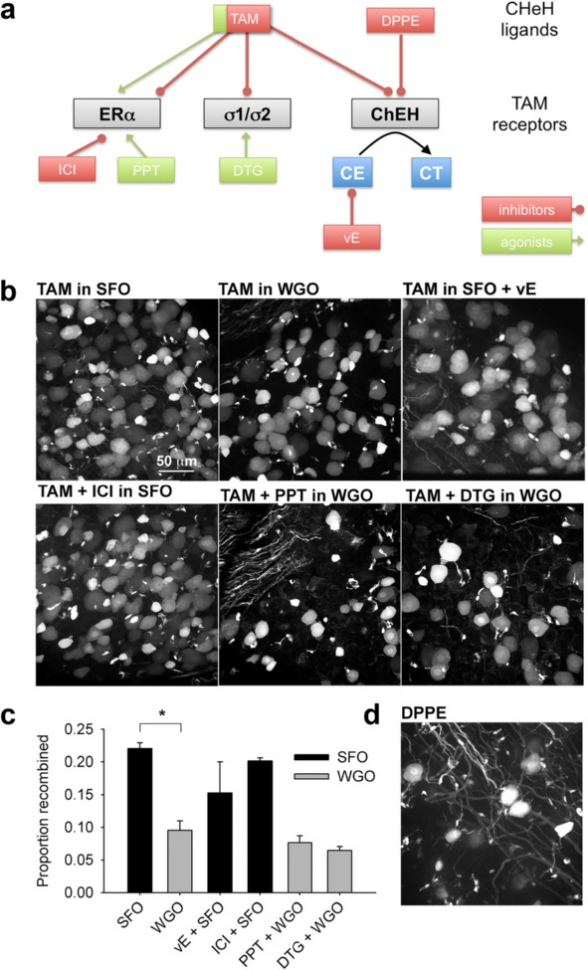
TAM induction of ATF3 expression occurs via ChEH inhibition. **a** Schematic indicating TAM targets (ERα: estrogen receptor alpha; σ1/σ2: sigma 1/2 receptors; ChEH: cholesterol-5,6-epoxide hydrolase), ChEH ligands (DPPE) and drugs used: ICI: ICI 182 780, a pure antiestrogen (*n* = 3); PPT: 4,4′,4″-(4-Propyl-[1*H*]-pyrazole-1,3,5-triyl)*tris*phenol, an ERα agonist (*n* = 3); DTG: ditolylguanidine, a sigma 1/2 receptor agonist (*n* = 3), vE: vitamin E. CE: ChEH substrate cholesterol epoxides; CT: ChEH products cholestane triol. **b** effects of SFO, WGO and drugs on recombination in the DRG. SFO: sunflower oil (*n* = 5); WGO: wheat germ oil (*n* = 4); vE: vitamin E (*n* = 6). **c** Only wheat germ oil significantly reduced recombination (*P* < 0.0001, one-way ANOVA). Note the decreased mean but increased variability in animals in which SFO was supplemented with vitamin E. **d** A single dose of DPPE, a selective ChEH ligand, induced significant recombination in the DRG

Next, we sought to determine by which mechanism TAM might induce cellular stress. We chose to use the DRG as a model since DRGs can be imaged rapidly as whole-mounts. TAM binds not only the mutated estrogen receptor (ERT2), but also endogenous ERα, the ChEH enzyme complex and the sigma1/2 receptors. TAM binding to ChEH inhibits the enzyme’s activity, resulting in the accumulation of cholesterol-5,6-epoxides and the inhibition of cholestane-3β,5α,6β-triol production [26]. To test the hypothesis that TAM-mediated accumulation of the ChEH substrate leads to Atf3 expression we took advantage of the fact that α-tocopherol (vitamin E) prevents the accumulation of cholesterol epoxides. Sunflower oil (SFO) contains approximately 40 mg/100 g α-tocopherol, while wheat germ oil (WGO) contains ~150 mg/100 g, nearly four times as much [15]. Therefore, we pre- and post-treated mice with i.p. WGO on the days before and after TAM treatment. We used two doses: either 0.25 ml or 0.5 ml, corresponding to 6.4125 or 12.825 IU/kg, and used it as the TAM vehicle (these non-toxic doses were chosen based on previous efficacy and toxicological studies of α-tocopherol in mice [16]). With either WGO schedule there was a drastic and statistically significant reduction in the number of recombined neurons in the DRG (Fig. 6a, b). We also attempted to deliver vitamin E directly in SFO, which was less successful at preventing TAM-induced Atf3 expression (Fig. 6c). This was likely due to increased variability as a result of the difficulties encountered when attempting to dissolve vitamin E evenly in SFO. Nevertheless, we found that the mean recombination frequency was consistently decreased in all tissues examined (dentate gyrus, olfactory bulb, and DRG, data not shown).

Finally, we asked directly whether activation of the ChEH complex can induce recombination in naïve ATF3-CreERT2:stop^fl/fl^tdtomato mice. We injected animals with a single intraperitoneal dose of the selective ChEH ligand DPPE (50 mg/kg) [21], and compared recombination to saline treated animals. A significant increase in tdtomato positive cells occurred (Fig. 6d and Table 2), supporting the idea that ChEH inhibition is responsible for TAM-induced *Atf3* upregulation. The overall density of recombined cells in DPPE-treated mice was far less than that of TAM (in SFO)-treated animals. This was expected, since DPPE cannot induce active nuclear translocation of the ERT2 construct and tdtomato induction is therefore entirely reliant on leaky nuclear translocation of CreERT2.

**Table 2:** DPPE, a selective ChEH ligand, significantly increased recombination in the DRG, hippocampus and olfactory bulb

Tissue	Saline	DPPE	*p* value	Analysis details
DRG	12.33 +/− 2.33	26.00 +/− 3.21	0.026	Neurons per DRG whole-mount, 2 cervical DRG/ n
Dentate gyrus	1.17 +/− 0.44	8 +/− 0.58	0.001	Granule cell neurons per section, 4 sections/ n, anterior dentate gyrus at the level of the median eminence
Olfactory bulb	2 +/− 0.58	12.33 +/− 1.45	0.003	Granule cell neurons per 150 × 500 micron field, mid-OB

Data represent mean +/− SE, *n* = 3 mice per group

Since some primary afferent neurons express ERα as well as the sigma-1 receptor [27, 28], we also assessed their putative roles in this context. We treated mice with either ICI 182,780, a “pure” antiestrogen [17], 4,4′,4″-(4-Propyl-[1*H*]-pyrazole-1,3,5-triyl)*tris*phenol (PPT), a potent ERα agonist [18], or ditolylguanidine (DTG), a sigma-1 and −2 receptor agonist. These drugs were given the day prior, on the day of, and the day following TAM treatment (75 mg/kg). None of these compounds induced recombination if given alone (data not shown), and none significantly altered the proportion of DRG neurons which underwent recombination (Fig. 6b). Drug doses and animal numbers are listed in Table 1.

**Fig. 7:**
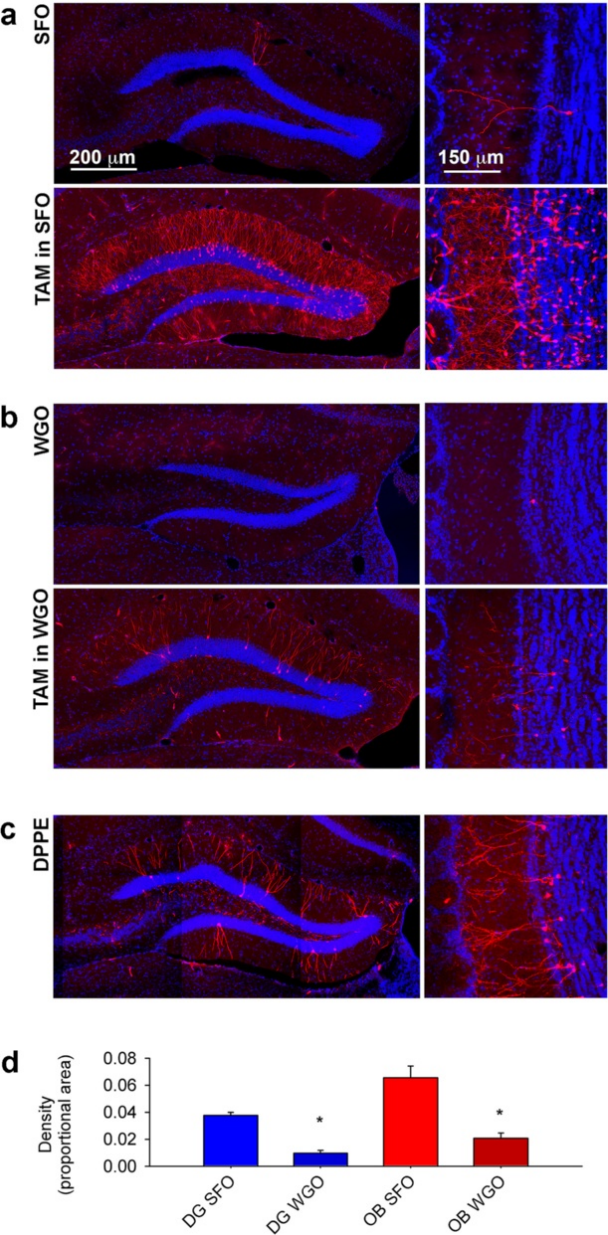
CreERT2-mediated recombination in dentate gyrus and olfactory granule neurons is abrogated by dissolving TAM in wheat germ oil (WGO) rather than sunflower oil (SFO). **a** tdtomato expression in dentate gyrus (left panels) and OB (right panels). **b** Administering TAM in WGO produces a dramatic reduction in tdtomato expression in both hippocampus and OB, as well as in vasculature and ependymal cells (not shown). **c** the effects of TAM are partially-recapitulated by DPPE, a selective ChEH ligand. **d** quantitative effects of TAM in sunflower oil (*n* = 5) compared to wheat germ oil (*n* = 4). All images are single 1 μm-thick optical confocal sections. OB: olfactory bulb

As was observed in the DRG, administration of TAM in WGO considerably decreased the ATF3 expression induced by the compound across most affected cell types and brain regions. Reduced recombination was particularly striking in the dentate gyrus of the hippocampus (Fig. 7a, b). Again, DPPE partially mimicked the effects of TAM in this area (Fig. 7c), suggesting an important role for ChEH inhibition in Atf3 activation. Quantification of these effects is provided in Fig. 7d and Table 2.

## Discussion

This is the first study to directly assess the effects of TAM on cellular stress-induced Atf3 expression in the nervous system. We show that granular neurons of the olfactory bulb and dentate gyrus, a subpopulation of sensory neurons, ependymal cells and endothelial and vascular smooth muscle cells upregulate Atf3 in response to a single 75 mg/kg dose of TAM. In addition, we show that TAM does not induce Atf3 via estrogen receptors or sigma 1/2 receptors in these cells. Instead, our results indicate that ChEH is the responsible TAM-binding entity: Atf3 induction is reduced when TAM is delivered in vitamin E rich wheat germ oil and, conversely, direct activation of ChEH using the selective ligand DPPE upregulates Atf3. Whether this is due to accumulation of cholesterol epoxides (ChEH ligands) or reduction in the synthesis of cholestane-3β,5α,6β-triol remains unknown, although the anti-cancer effects of TAM in estrogen-insensitive breast tumors are thought to be attributable to the former [11, 26, 29].

In the cortex, we found TAM to induce recombination in granular neurons of the dentate gyrus of the hippocampus and olfactory bulb, as well as in vascular and ependymal cells. Taking previous findings and our results at face value, they would suggest that recombined neurons in the dentate gyrus occupy relatively superficial positions in the granular cell layer. Both during development and in adulthood, dentate granule neurons are born in an outside-out manner [8, 30], implying that those which are most susceptible to TAM-induced Atf3 upregulation are more mature cells.

A previous investigation of TAM-induced toxicity at therapeutic doses in the brain revealed a loss of oligodendrocyte progenitor cells (OPCs) in the corpus callosum [7]. This was accompanied by a reduction in their proliferation, and that of neural progenitors in the subventricular zone and dentate gyrus, and it was suggested that this may represent a cellular substrate of cognitive dysfunction experienced by breast cancer patients receiving TAM therapy. OPC loss was prevented both in vitro and in vivo by MEK1/2 inhibition, one of a number of intracellular messengers that can activate Atf3 expression [12]. In contrast, we found no evidence of TAM-induced recombination in OPCs or in the deeper neurogenic regions of the dentate gyrus or subventricular zone. It is possible that the reported OPC loss occurs independent of Atf3 induction, and although our study does not rule out effects of TAM on neurogenesis, it does not support a role for Atf3 in this process. Together these data indicate pleiotropic effects of TAM on dentate granule neurons, one involving MEK1/2 activation in immature neural cells (neuronal and glial progenitors), the other reliant upon Atf3 upregulation induced by ChEH inhibition in older adult-born neurons.

One point to take away from our studies in particular is that patients prescribed tamoxifen take the medication on a daily basis. We found that a single tamoxifen dose induced ATF3 expression, albeit transiently, in several populations of neurons. Our data show that ATF3 expression can be maintained in susceptible neurons with repeated daily dosing (Fig. 4), the downstream effects of which are unknown, but likely to be of biological and clinical significance.

TAM also induced Atf3 upregulation in sensory neurons, which was particularly marked in large proprioceptive NF200 positive cells in the trigeminal mesencephalic nucleus and in those projecting to Clarke’s column in the spinal cord, and to a much lesser extent in non-peptidergic IB4 positive neurons. It is tempting to speculate that large fibres have higher metabolic demands and might therefore be more vulnerable to cellular stress. The pattern of induction is certainly evocative of a large fibre neuropathy, which is known to accompany non-hormonal chemotherapy with platinum-based drugs [31] and has been linked to mitochondrial dysfunction. However, with the exception of rare optic neuropathies [32, 33], we can find no evidence in the literature that TAM treatment results in any sensory disturbances. This could be because Atf3 in the peripheral nervous system is associated with a robust reparative response after stressors such as axotomy. *Atf3* is among the earliest genes to be upregulated after axonal injury [34], and is associated with successful regeneration: it is robustly induced in regeneration-competent sensory neurons by peripheral nerve injury [35] but not by injury to centrally-projecting dorsal roots [36]. Furthermore, genetic overexpression of *Atf3* in sensory neurons improves the regenerative response to peripheral nerve injury [37, 38].

Beyond its therapeutic use in breast cancer, TAM has been exploited experimentally to induce permanent reporter expression in, for example, newly-born neurons in the olfactory bulb and dentate gyrus [8], in physiologically-active neurons throughout the brain [9], and in progeny of ependymal cells of the intact and injured murine spinal cord [39]. In each of these examples, the use of TAM to induce recombination may have profound consequences on interpretation of the data.

In the Imayoshi study [8], very large doses (400 mg/kg) were administered daily for 4 days, a regimen that was repeated twice more in some experiments. What is absent, however, both in anatomical and behavioural experiments assessing the role of newly-generated hippocampal neurons in memory formation/retention, is a TAM-only control in wild-type mice. Based on our results, TAM treatment would (repeatedly) induce cellular stress in older neurons, the effect of which on the neurogenic process remains unknown.

The Guenthner study [9] used a genetic labeling technique nearly identical to ours, but recombination was driven by promoters for the immediate-early genes *Fos* and *Arc*, upregulated as part of the “excitation-transcription” neuronal response to synaptic activity initiated by CREB phosphorylation [40]. TAM-induced recombination in the dentate gyrus and olfactory bulb, particularly when recombination was dependent upon Arc expression, was remarkably similar to what we show here (Figs. 5 and 7), both in terms of number of neurons recombined and dorso-ventral distribution in the granular cell layer of the dentate gyrus. In these regions particularly it is unclear to what extent neuronal activity *per se* was driving recombination, or whether TAM induced Arc expression via cellular stress as it does expression of Atf3.

Our data show that ependymal cells are particularly prone to TAM-induced Atf3 upregulation. This may have important ramifications for the interpretation of a study by Barnabé-Heider et al. [39]. Here, five daily treatments of TAM (80 mg/kg) were used to induce recombination in ependymal cells in FoxJ1-CreER mice in order to determine whether ependymal cells are multipotential in vitro, and in vivo following a spinal cord injury. The in vitro studies showed that 3 days after cessation of TAM treatment, recombined ependyma could indeed give rise to neurospheres with cells expressing markers for neurons, astrocytes and oligodendrocyte-lineage cells. In vivo, reporter-expressing ependyma self-renewed and produced astrocytes and oligodendrocyte-lineage cells 5 days following the last TAM dose. Again, what remains unknown is the effect that TAM-induced cellular stress has on the multipotentiality of ependymal cells, and thus on the interpretation of the results.

In all three of the above studies, TAM was delivered in corn oil, which has a vitamin E content of ~14.3 mg/100 g, compared to ~40 mg/100 g in SFO and ~150 mg/100 g in WGO. ChEH inhibition, which is complete even at lower (therapeutic) doses of TAM [11], is thus likely to have contributed significantly to the observed effects.

## Conclusions

The ATF3-CreERT2 mice we have generated have allowed the first glimpse into the cellular stress pathway elicited in the central nervous system by TAM. They have revealed that TAM induces Atf3 expression in CNS and PNS neurons, vasculature and ependyma by interfering with cholesterol biosynthesis, an important issue when considering the use of ChEH inhibitors (such as DPPE) in the treatment of cancer. They also give cause for caution when interpreting experiments using the Cre-ERT2 system to manipulate gene expression. Our finding that vitamin E prevents TAM-induced cellular stress represents a solution to this previously unappreciated experimental confound.
